# Multivariate Curve Resolution and Carbon Balance Constraint to Unravel FTIR Spectra from Fed-Batch Fermentation Samples

**DOI:** 10.3390/bioengineering4010009

**Published:** 2017-01-25

**Authors:** Dennis Vier, Stefan Wambach, Volker Schünemann, Klaus-Uwe Gollmer

**Affiliations:** 1 AG Biophysik und Medizinische Physik, Technische Universität Kaiserslautern, Kaiserslautern 67663, Germany; schuene@physik.uni-kl.de; 2 Fachbereich Bioverfahrenstechnik, Hochschule Trier, Umwelt-Campus Birkenfeld, Birkenfeld 55761, Germany; s11a81@umwelt-campus.de; 3 Fachbereich Angewandte Informatik, Hochschule Trier, Umwelt-Campus Birkenfeld, Birkenfeld 55761, Germany; k.gollmer@umwelt-campus.de

**Keywords:** multivariate curve resolution, *E. coli*, fed-batch, fermentation, carbon mass balance constraint, soft constraints, alternating least squares, hybrid modelling

## Abstract

The current work investigates the capability of a tailored multivariate curve resolution–alternating least squares (MCR-ALS) algorithm to analyse glucose, phosphate, ammonium and acetate dynamics simultaneously in an *E. coli BL21* fed-batch fermentation. The high-cell-density (HCDC) process is monitored by ex situ online attenuated total reflection (ATR) Fourier transform infrared (FTIR) spectroscopy and several in situ online process sensors. This approach efficiently utilises automatically generated process data to reduce the time and cost consuming reference measurement effort for multivariate calibration. To determine metabolite concentrations with accuracies between ±0.19 and ±0.96·gL^−l^, the presented utilisation needs primarily—besides online sensor measurements—single FTIR measurements for each of the components of interest. The ambiguities in alternating least squares solutions for concentration estimation are reduced by the insertion of analytical process knowledge primarily in the form of elementary carbon mass balances. Thus, in this way, the established idea of mass balance constraints in MCR combines with the consistency check of measured data by carbon balances, as commonly applied in bioprocess engineering. The constraints are calculated based on online process data and theoretical assumptions. This increased calculation effort is able to replace, to a large extent, the need for manually conducted quantitative chemical analysis, leads to good estimations of concentration profiles and a better process understanding.

## 1. Introduction

Multivariate curve resolution (MCR) with constrained alternating least squares (ALS), as described by Tauler et al. [1], is a powerful method to deconvolve overlapping spectral signals from chemical and biological reaction systems. The intended purpose is commonly the estimation of concentrations of individual components **C** or the identification of unknown spectral profiles **S** in complex aqueous solutions; generally, MCR has the ability to estimate both simultaneously from a data matrix **X**. The specific feature of MCR is the decomposition of **X** in a physically or chemically meaningful way. Besides MCR and ALS, other bilinear modelling methods and different algorithms can be utilised for the decomposition of **X**, with various resolution performances and limitations [2]. Because of its flexibility and popularity [3], MCR with the ALS algorithm is used in this work. A tutorial for the application of MCR to analyse multicomponent systems, with special focus on the ALS algorithm, is given in [4]. The main aspects to be considered—such as data set configurations, initial estimates and applicable constraints—are described. A central issue of bilinear decomposition is the impact which particular constraints, initial estimates and the applied algorithm may have on the uniqueness of solutions in the presence of rotational ambiguities [5]. In the present work, the initial MCR settings and constraints for the analysis of FTIR-spectra from an *E. coli* fed-batch bioprocess are described in detail.

Compared with other established chemometric analysis methods, MCR has the potential of simultaneous resolution and quantitation of all mixture components without their chemical or physical separation [5,6]. Besides the recovery of qualitative and quantitative information about analytes, the identification of unknown interferents is possible [7]. In comparison to other multivariate calibration methods, the calibration effort of MCR can be decreased significantly by application of appropriate constraints [8]. Given a suitable set of constraints, this paper demonstrates that single measurements of pure solved analytes suffice to perform quantitative MCR analysis of respective fermentation data.

MCR-ALS has been employed for years in different research fields, especially in chemical reaction processes monitored by different spectroscopic techniques such as X-ray absorption [9], fluorescence, nuclear magnetic resonance, Raman, Near-Infrared and FTIR [10]. In biochemical and biophysical processes, MCR was used to analyse protein and nucleic acids systems concerning denaturation processes, protonation equilibria or complexation processes [11]. Among other chemometric methods, MCR was utilised in single biological cell analysis to unmix information from hyperspectral images [12]. In reference to fermentation processes, several applications of MCR-ALS have been published, e.g., in monitoring alcoholic fermentations with *S. cerevisiae* [13,14], milk lactic acid fermentations with *Streptococcus* and *Lactobacillus* strains [15] and the quantification of penicillin V in bioprocesses with *Pencillium chrysogenum* [16]. In this work, MCR-ALS with tailored constraints is applied to estimate metabolism-relevant concentrations in high cell density cultivation (HCDC) fed-batch processes with *E. coli* BL21 (DE3) pET28a. The cultivated organism produces the recombinant and pharmaceutically utilisable enzyme cytochrome p450 after induction. For evaluating process kinetics and optimising the growth of microorganisms, it is useful to obtain estimations about the quantitative changes of carbon, nitrogen and phosphate sources as well as of metabolic products such as acetic acid in the fermentation broth over process runtime. The aim of this study is the resolution of these substances by a tailored MCR-ALS algorithm. 

To monitor the composition of fermentation media, ATR-FTIR spectroscopy is employed as an in-line ex situ analyser. Spectral information from the fermentation process is provided online by automatic cell-free sampling of fermentation broth through an ATR flow-cell. Because of the continuous sterile sampling, the constraint of invariance of the total concentration (closure) is applicable, as described below. An automated in-line flow system can cause the problem of CO_2_ and air bubbles, as well as biofilms on the ATR surface [17]. Therefore, biofilms are inhibited by employing cell-free sampling and initial ethanol-cleaning of the flow-system. As technical gas bubble prevention, just gas-tight polytetrafluoroethylene (PTFE) tubes are implemented. However, principally, the problem of gas bubbles can be handled mathematically by the MCR algorithm as shown in this study.

FTIR spectroscopy is an established technique in bioprocess monitoring and, as other IR techniques such as near-infrared (NIR) and Raman, it combines the advantages of non-invasiveness and fast simultaneous measurement of multiple solved substances [18]. An overview of advantages and disadvantages of different spectroscopy techniques in bioreactor monitoring has been described [19]. In the mid-infrared region, covered by FTIR, most excitations of fundamental molecular vibrations can be found. Especially the fingerprint area (1500–500 cm^−1^) exhibits specific patterns of media compounds [20]. By contrast, the peaks in the NIR spectrum consist of overtones and combinations from primary MIR signals and are less distinctive. In comparison to NIR, the MIR region exhibits a higher selectivity thus allowing for a better detection of overlapping component spectra in complex aqueous mixtures. Raman spectroscopy is not sensitive to water and the small peak widths of solved components are main advantages of this technique in bioprocess monitoring [21]. However, the Raman scattering is generally weaker than the FTIR signal, while higher concentrations of target analytes are required. A comparison of FTIR, NIR and FT-Raman spectroscopic techniques referring to a lactic acid fermentation shows the best prediction performance for FTIR [22].

Some studies described the analysis of glucose, acetate, ammonium and phosphate concentrations in bioprocesses, using ATR-FTIR spectroscopy. Among other substances, ammonium and glucose are analysed in a complex antibiotic fermentation by at-line measurements on a horizontal attenuated total reflectance (HATR) crystal using a partial least squares (PLS) calibration model [23]. Gluconacetobacter xylinus fed-batch cultures were monitored by an in situ ATR probe aimed at the online PLS analysis of acetate, phosphate and ammonium [24]. The results of these PLS predictions—in contrast to MCR-ALS predictions of glucose, acetate, ammonium and phosphate—are discussed below.

As shown by references, FTIR monitoring of bioprocesses and MCR analysis of complex mixtures such as fermentation broths promise many advantages in simultaneous process information collection. The proposed effective usage of in situ and ex situ online sensor data to calculate carbon mass balance-constrained MCR predictions of several analytes underlines the relevance of ATR-FTIR/MCR-ALS combinations in fermentation analysis.

The monitored substances are related to bacterial metabolism. Glucose, ammonia and phosphate are substrates whereas acetate is a by-product of overflow metabolism [25]. The recombinant cytochrome p450 remains unconsidered for its being an intracellular metabolite and therefore not being obtainable in fermentation broth. To predict concentrations of the observed analytes, only four calibration measurements of pure components are required, provided adequate constraints are applied during the alternating least squares procedure. In addition to the required analyte spectra, the implementation of estimated artefact spectra and additionally known fermentation media components is useful. As mentioned above, collecting samples from fermentation broth by a peristaltic pump through a tube system can present the problem of air bubbles in ATR flow-cell with impact on the measured spectra. If the water spectrum is removed from each mixture spectrum prior to that, the air bubble disturbance has its own spectral signature and can be handled like any pure component in multivariate curve resolution. The shape of this artefact is easy to be determined and its implementation in MCR improves the resolution of primary signals, as shown below. All initial estimations for pure spectral components **S** expected in the mixture are also implemented as soft-constraints during alternating least squares. In the following, soft-constraints means the presence of an allowed solution area in a range set by inequality constraints, as in the optimisation problem. In the case of physical spectral shifting in the mixture, a certain flexibility during iterative identification of pure components helps to avoid over-restriction. 

In the concentration estimation step, besides the non-negativity constraint, online process data such as input and exhaust gas-flow, fermenter mass, liquid supply from feed reservoir and pH control as well as turbidity are utilized to calculate elementary carbon mass balance constraints. Mass balance constraints (closure) applied to reaction systems have been described [1,26,27]. Closure constraints require invariance of total concentration, granted by the sterile online sampling system and by including total reactor in- and output mass flow in the constraint calculation. So far, as known, the presented application of the carbon mass balance constraint for MCR to analyse a fed-batch fermentation process is a new utilisation of the popular closure constraint. The carbon mass balance constraint for a fed-batch fermentation requires extensive prior calculations, such as different conversion steps and soft sensor approaches. In addition, dynamic in- and output carbon mass-flow in gas and liquid phase as well as continuous and discrete sampling need to be taken into account. The presented algorithm is able to deal with these requirements. Calculating carbon mass balances or recovery rates is an established approach in bioprocess engineering to check the integrity of observed process data [28]. The referenced literature has already shown the application of carbon mass balances for *Escherichia coli* high-cell-density fed-batch culture and recombinant protein production. During process runtime, carbon recovery rates should take on values of about 1. In the present study, this condition is utilised as a MCR-ALS constraint for the estimation of carbon sources and metabolites such as glucose and acetate in fermentation media. In so doing, the explorative decomposition of measured mixture spectra is coupled with analytical knowledge in order to form a new hybrid multivariate modelling approach.

In summary, the objectives of this paper are

(1) the interpretation of the MCR-ALS closure constraint as a carbon mass balance constraint for fed-batch fermentation processes;
(2) to demonstrate that moderate gas bubble disturbances on the ATR crystal can be handled computationally, without any need for technical preventions;
(3) to show that MCR-ALS with carbon mass constraint is capable of simultaneously predicting four analyte concentrations from FTIR spectra of fermentation media samples, with minor calibration effort.

## 2. Material and Methods

### 2.1. Spectra Acquisition, Sampling and Spectra Processing

The MIR spectra are scanned with a Thermo Scientific Nicolet™ iS™ 10 and the extension unit Nicolet iZ™ 10. The Specac’s Gateway™ ATR Accessory Kit and a ZnSe ATR crystal with six reflections are mounded as a flow cell in that unit. The flow cell is connected to the bioreactor via PTFE tubes (id 1.1 mm) and a Flownamics^®^ FISP^®^ probe with rapid flow membrane for cell-free sampling. A peristaltic pump, controlled by an Arduino microcontroller and a driver board, delivers the sample liquid continuously to the FTIR flow cell. A background spectrum with pure water in the flow cell is scanned before using the FTIR for bioprocess analysis. During a running sampling process, spectra are scanned in cycles of 10 min. During spectrum acquisition by the Thermo Scientific™ OMNIC™ software [29], the sampling pump remains inactive. The spectra acquisition time for scanning 32 spectra and releasing the mean spectrum for the current sample is about 1 min. OMNIC and microcontroller are both triggered by a C# program that observes and synchronises the sample supply and measurement steps. Before each start of a fermentation trial, tubing and flow cell are treated with 70% ethanol solution to minimise the risk of microbial activity in the sampling section. The initial spectra of known substances are standardised to unit concentration. No further pre-processing steps such as normalisation or differentiation are applied to the mixture-spectra in order to preserve the natural physical properties of the spectra. After the fermentation run, the MCR-ALS analysis of the ex situ online-monitored FTIR spectra is performed for all collected spectra.

### 2.2. Reference Analysis

To validate MCR-ALS results, reference values for glucose, acetate, ammonia and total phosphate concentrations are measured in the cell-free sample drain after passing the FTIR flow-cell. Glucose analysis was conducted by the YSI 2700 SELECT Biochemistry Analyzer (Yellow Springs, OH, USA). Acetate was determined by HPLC (high performance liquid chromatography) using chromatography column Reprogel H+ (Dr. Maisch GmbH, Ammerbuch, Germany). Total phosphate and ammonia were determined using photometric methods by procedures described in DIN EN 1189, DVGW W 504 and DIN 38406 E5.

### 2.3. Bioreactor System and Online Measurement Equipment

Fermentations are conducted in a prototype of Bioengineering’s 5l rounded-bottom autoclavable laboratory fermenter (RALF), controlled and observed with the Software BioSCADA Lab (Bioengineering AG, Wald, Switzerland). A supply tower with intelligent front modules (IFM) directs in- and output of control and measurement values. All data interchanged between IFMs and SCADA pass a structured query language (SQL) data base, the central data hub. From there, the needed data for calculating MCR constraints or advanced measurement and control strategies can be acquired by MySQL and MATLAB.

The current work utilises the following bioprocess online measurement instrumentation: Turbidity probe ASD19-N and optek-converter FC10 (optek-Danulat GmbH, Essen, Germany); exhaust gas analyser BlueInOne Ferm (BlueSens GmbH, Herten, Germany); thermal mass flow controller Red-Y Smart for inflow oxygen (0.01, …, 5 lpm) and air/nitrogen (0.1, …, 10 lpm) control (Vögtlin Instruments AG, Aesch, Switzerland); balances for online weight/volume observation of fermenter (DE 35K5D, Kern & Sohn GmbH, Balingen,Germany), acid/base (EW6000-1M, Kern & Sohn GmbH, Balingen, Germany) and feed (BL6100, Sartorius, Göttingen, Germany) reservoir.

### 2.4. Fermentation Strategy

The HCDC process is conducted in three phases: an initial batch phase, a feeding phase for biomass growth and an induction phase for product expression. The substrate and inductor feed is performed by exponential feeding strategy to control the cell specific growth rates µ similar to [30]. Because of the risk of overflow metabolism und protein folding errors at high growth rates, µ is controlled to defensive setpoints of 0.1 h^−1^ (feed phase/biomass production) and 0.05 h^−1^ (induction phase).

### 2.5. Strain and Fermentation Medium

*E. coli* BL21 (DE3) pET28a was stored as glycerol cryo-culture at −76 °C. The pre-culture is incubated as overnight culture in 500 mL baffled flasks at 37 °C in a shaker rotating 200 rpm. An amount of 300 mL pre-culture is portioned in equal shares on two shaking flasks. After 24 h pre-culture incubation, 2.7 L sterilised batch medium in the reactor is inoculated with the culture, thus amounting to a total start volume of 3 L.

The media are modified mineral media based on [31]. The pre-culture and batch medium contain per litre: Glucose*H_2_O, 16.5 g; KH_2_PO_4_, 13.3 g; (NH_4_)_2_HPO_4_, 4 g; citric acid, 1.7 g; MgSO_4_*7H_2_O, 0.72 g; Fe(II)SO_4_*7 H_2_O, 113.5 mg; CoCl_2_*6H_2_O, 10.5 mg; MnCl_2_*4 H_2_O, 15 mg; CuCl_2_, 1.2 mg; H_3_BO_3_, 3 mg; Na2MoO4*2 H2O, 2.5 mg; thiamine*HCl, 4.5 mg; trisodium citrate dihydrate, 75 mg; Na_2_-EDTA, 9.6 mg.

The feeding solution is composed of Glucose*H_2_O, 544.4 g; MgSO_4_*7H_2_O, 12 g; Fe(II)SO_4_*7H_2_O, 43.3 mg; CoCl_2_*6H_2_O, 21.4 mg; MnCl_2_*4H_2_O, 23.5 mg; CuCl_2_ 2.5 mg; H_3_BO_3_, 5 mg; Na_2_MoO_4_*2H_2_O, 4 mg; trisodium citrate dihydrate, 116 mg; Na_2_-EDTA, 14.8 mg.

## 3. Theory and Calculation

### 3.1. Nomenclature

Matrices: Uppercase fat letters 

Vectors: Lowercase fat letters

Scalars: Lowercase letters

### 3.2. Multivariate Curve Resolution and Its Physical Interpretation

The bilinear model of multivariate curve resolution [1] for FTIR data can be deduced from the Lambert–Beer law which describes the attenuation of light travelling through material. The absorbance x of a material is given as

$$x\;=\mathrm{lg}\left(\frac{I_{0}}{I_{1}}\right)=c\;\varepsilon \;d$$

The logarithm of incident radiant intensity (I_0_) divided by transmitted radiant intensity (I_1_) is equal to the product of substance concentration (c), the molar attenuation coefficient (ε) and the pathlength (d). In this work, the technique of attenuated total reflection is used, so d is the penetration depth of an evanescent wave into the sample on the ATR crystal. The material and wavelength dependent factors ε and d can be pooled to s which consolidates the optical properties of a substance:

x = c s

For mixtures of several substances k = 1, …, Ω, each absorbance value x_ij_ related to its wavelength in a spectrum j = 1, …, n for a particular concentration profile i = 1, …, m is calculated as

$$x_{\mathrm{ij}}=\overset{Ω}{\underset{k=1}{\sum }}c_{\mathrm{ik}}{\;\varepsilon }_{\mathrm{kj}}d_{\mathrm{kj}}=\overset{Ω}{\underset{k=1}{\sum }}c_{\mathrm{ik}}s_{\mathrm{kj}}\;$$

In chemometrics, it is usual to term i = 1, …, m as the objects or samples of a dataset, whereby j counts the n features or variables. Here, the m objects are samples of fermentation broth over process runtime and the n features are absorbance values over the wavenumbers of FTIR spectra.

According to the previous sum equation, the decomposition of absorbance values over sample and wavenumber can be organised in matrices

$$\left(\begin{array}{cccc} x_{11} & x_{12} & \cdots & x_{1n}\\ x_{21} & x_{22} & \cdots & x_{2n}\\ \vdots & \vdots & \ddots & \vdots \\ x_{m1} & x_{m2} & \cdots & x_{\mathrm{mn}} \end{array}\right)=\left(\begin{array}{cccc} c_{11} & c_{12} & \cdots & c_{1Ω}\\ c_{21} & c_{22} & \cdots & c_{2Ω}\\ \vdots & \vdots & \ddots & \vdots \\ c_{m1} & c_{m2} & \cdots & c_{mΩ} \end{array}\right)\left(\begin{array}{cccc} s_{11} & s_{12} & \cdots & s_{1n}\\ s_{21} & s_{22} & \cdots & s_{2n}\\ \vdots & \vdots & \ddots & \vdots \\ s_{Ω1} & s_{Ω2} & \cdots & s_{Ωn} \end{array}\right)$$

In matrix representation, we get the simplified description:

$$X=CS^{T}$$

That is the decomposition of absorbance spectra indicated by multivariate curve resolution assuming the data matrix **X** is bilinear.

### 3.3. An Implementation of the Alternating Least Squares Algorithm

With an initial estimation for concentration matrix ${\hat{C}}_{0}$ or pure components ${\hat{S}}_{0}$ and existing data **X,** the ALS algorithm can run and perform multivariate curve resolution iteratively [1]. Assuming the chemical rank of the observed data matrix is estimated and one assumption per each expected spectral independent component is available, the ALS procedure can start with an initial pure component matrix. Thus, in the first iteration, the estimated unconstrained concentration matrix $\hat{C}$ is obtained by

$$\hat{C}=X{\hat{S}}_{0}{\left({\hat{S}}_{0}^{T}{\hat{S}}_{0}\right)}^{-1}=X{\left({\hat{S}}_{0}^{T}\right)}^{+}$$

whereby the superscripted + indicates the pseudoinverse.

In the next step, ${\hat{S}}^{T}$ is estimated in an unconstrained way by

$${\hat{S}}^{T}={\left({\hat{C}}^{T}\hat{C}\right)}^{-1}{\hat{C}}^{T}X={\hat{C}}^{+}X$$

With that pure component estimation, a new concentration matrix calculation can be performed. That loop is repeated until a termination criterion is achieved.

Because of rotational and intensity ambiguities, it is necessary to constrain the solutions for $\hat{C}$ and ${\hat{S}}^{T}$ to obtain a physically meaningful separation of mixture components.

To calculate constrained linear least-squares solutions in this work, the lsqlin function with the *active-set* algorithm from MATLAB and the “Optimization Toolbox” is applied [32]. lsqlin makes use of mathematically rigorous methods of applying equality and inequality constraints with a better numerical stability than approximate methods commonly used in chemometrics. The approximate methods are easy to use and code, but they exhibit poor least squares behaviours and in some cases they result in an increase in the magnitude of residuals [33].

lsqlin solves linear least-squares curve fitting problems of the form

$$\underset{c^{T}}{\min}∥x^{T}-S\;c^{T}{∥}_{2}^{2}\;\mathrm{such}\;\mathrm{that}\;\{\begin{array}{c} Ac^{T}\leq b\\ \begin{array}{c} A_{\mathrm{eq}}c^{T}=b_{\mathrm{eq}}\\ l\leq c^{T}\leq u \end{array} \end{array}$$

**Figure 1:**
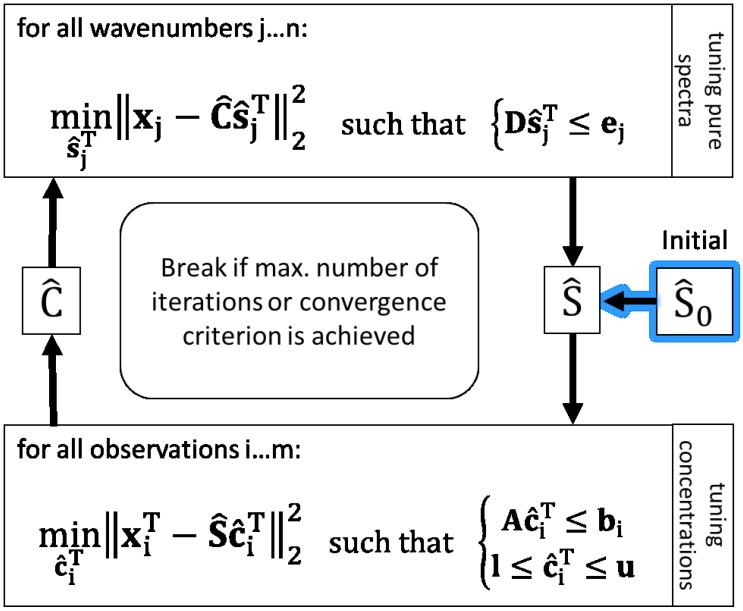
Scheme of the MCR-ALS algorithm with inequality constraints applying MATLAB lsqlin function.

Hence, the MCR-ALS algorithm using lsqlin is implemented as shown in Figure 1 to solve the present problem of resolving **X** in a hybrid modelling way with the target of reducing ambiguities of least squares solutions. To bring a priori knowledge about pure spectra and the bioprocess into ALS solutions, linear inequality constraint vectors (e.g., **b**) and matrices (e.g., **A**) are applied. Further, the non-negativity constraint for concentrations is set by using lower bounds (**l**).

### 3.4. Constraints for Pure Spectral Component Estimation

Pure spectra of components which are known and expected in mixture are constrained based on measured und normalised spectra of respective pure substances. Therefore, the same spectra used as initial estimations ${\hat{S}}_{0}$ are also basis values of inequality constraints to calculate $\hat{S}$. Assuming the shapes of pure spectra in mixture closely resemble the pure measured spectra, in each iteration the associated pure component estimations may only vary inside the defined ranges relative to the measured ${\hat{S}}_{0}$**.** Depending on the amount of expected deviation in mixture, the range for the respective component can be adapted. In so doing, over-restriction can be avoided e.g., in the case of smaller rates of band shifting or in the case of differences in signal-to-noise ratios between high concentrated pure substance measurement and lower concentrations in mixture.

Regarding the inequation constraint for tuning $\hat{S}$ in Figure 1 (upper box), **D** is composed vertically of the positive and negative identity matrices **I** and −**I** both of dimension (Ω, Ω).

$$D=\left(\begin{array}{c} +I\\ -I \end{array}\right)$$

The positive part is associated with the upper bounds $e_{j}^{u}$, the negative with the lower bounds $e_{j}^{l}$ represented in **e**_j_ for all components on each wavenumber. The allowed upper deviation **u** and lower deviation **l** are relative to the total ranges of the minimal and maximal values of the pure initial spectra for each component ${\left({\hat{s}}_{0}\right)}_{k=1,\ldots ,\Omega }$.

$$\Delta_{s}=\left(\begin{array}{c} \max({\left({\hat{s}}_{0}\right)}_{k=1})\\ \vdots \\ \max\left({\left({\hat{s}}_{0}\right)}_{k=\Omega }\right) \end{array}\right)-\left(\begin{array}{c} \min({\left({\hat{s}}_{0}\right)}_{k=1})\\ \vdots \\ \min\left({\left({\hat{s}}_{0}\right)}_{k=\Omega }\right) \end{array}\right)$$

$$e_{j}=\left(\begin{array}{c} +e_{j}^{u}\\ -e_{j}^{l} \end{array}\right)=\left(\begin{array}{c} {\left({\hat{s}}_{0}\right)}_{j}^{T}+u\circ \Delta_{s}\\ {\left({\hat{s}}_{0}\right)}_{j}^{T}-l\circ \Delta_{s} \end{array}\right)$$

In our application, the chemical rank of the mixtures **X** was estimated at 12 significant spectroscopically independent components by principal component analysis (PCA). The loadings of PCA were manually evaluated for the presence of spectra-like structure, which is strongly present on the first principal components and decreases on higher factors. Among the above mentioned, significant spectroscopically independent components were spectra of known media components, expected metabolic products, artefacts (like air bubbles) and unknown components. Only components evaluated as certainly present in the spectral mixture **X** are constrained, notably the pure spectra of wanted substances: glucose, ammonia, total phosphate (H_2_PO_4_**^−^** + HPO_4_^2**−**^) and acetate. The estimations for those pure components may take on values at an interval of ±10% in the range of each pure component spectrum starting on the initial spectrum (see Table 1). 

Because of water background subtraction on each taken spectrum **x**_i_, air bubbles in the flow cell have the shape of inverted water spectra. Moreover, a pure water spectrum is also initialised for the case of air bubble presence during the background recording. Both estimations are not constrained and can vary depending on the actual mixture content.

**Figure 2:**
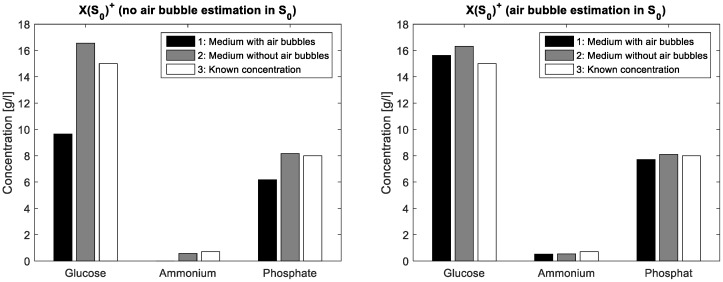
Proof of the initial estimation for the spectral air bubbles signature: in a known aqueous solution of glucose, ammonium and phosphate, air bubbles are present in one case and absent in another. On the left, the known spectral components S_0_ do not consider the air bubble model. On the right, the air bubble model is integrated and the differences between concentrations estimated in medium with and without air bubbles are similarly close to the known concentrations.

To demonstrate the validity of the assumption for the spectral air bubble model, a simple aqueous solution containing glucose (15·gL^−1^), ammonium (0.7 gL^−1^) and phosphate (8·gL^−1^) was compounded. From this solution, a first FTIR spectrum was acquired from a mixture covering the entire ATR crystal whereas a second spectrum resulted from the same mixture covering only about half of the crystal surface. In this way, a part of the IR beam reflections interacts with the aqueous solution on the ATR crystal, while another part interacts just with the air on the crystal surface. The latter liquid-free surface part simulates a large air bubble on the crystal in ATR flow cell. In the case without ALS iterations, the mixture matrix of the known solution is multiplied once with a simple pseudo inverse of the estimated initial pure components matrix. In a first measurement, S_0_ contains just pure measurements of glucose, ammonium and phosphate, respectively standardised to unit concentration. Next, S_0_ additionally contains an inverted water spectrum. The differences in concentration estimations are shown in Figure 2 whereas the actual concentration values are listed in Table 2. Obviously, the integration of the air bubble model brings an improvement of the prediction results in the case of air bubble presence.

### 3.5. Constraints for Concentration Estimation

In bioprocess engineering, the carbon balance and recovery rate of a fermentation process are commonly used as a check for the integrity of process monitoring and sensors as well as the assessment of the release of outer membrane components. Carbon balances in a fed-batch culture are based on the mass of carbon in the total fermenter volume. Thereby, the recovery rate is the relation between the recovered carbon m^C,rec^(t) and the carbon brought into the bioreactor m^C,in^(t) over process runtime t.

$$r^{C}\left(t\right)=\frac{m^{C,\mathrm{rec}}\left(t\right)}{m^{C,\mathrm{in}}\;\left(t\right)}$$

Suppose all carbon compounds are determinable and measurement errors are negligible, r^C^(t) is equal to 1 for all t. Because of the presence of measurement errors and not identified soluble organic carbon compounds, a tolerance range must be assumed. The carbon recovery considering biomass, CO_2_, glucose and acetate is assumed as being about 90% [28].

The recovered carbon is the sum of carbon mass in the reactor liquid phase L, gas phase g, sample liquid phase divided in cell-free sampling scf and cell containing sampling scc.

$$m^{C,\mathrm{rec}}\left(t\right)={\;m}^{C,L}\left(t\right)+m^{C,g}\left(t\right)+m^{C,\mathrm{scc}}\left(t\right)+m^{C,\mathrm{scf}}\left(t\right)$$

The brought-in carbon is the sum of initial carbon mass in fermentation medium at process start time (t = 0) and the supplied carbon mass m^r^ from the feed reservoir r.

$$m^{C,\mathrm{in}}\left(t\right)={\;m}^{C,L}\left(t=0\right)+m^{C,r}\left(t\right)$$

If the integrity of measurement equipment and data observation is already proved, the carbon mass balance can be applied as a MCR-ALS constraint for glucose and acetate estimation from the spectra on each observation i over process runtime. For that, several non-spectroscopic measurements and assumptions must be applied to calculate carbon balances on each FTIR measurement.

The carbon in the reactor liquid phase is located in biomass in fractions of $\alpha^{C,\mathrm{cell}}$ as well as in dissolved CO_2_ in fractions of $\alpha^{C,{\mathrm{CO}}_{2}}$. The fraction of carbon in biomass is an assumption based on the analysis of elemental biomass composition of *E. coli* with an elemental analyser taken from literature [34]. Further carbon, of course, is located in glucose (glc) and acetate (ace), for which the concentrations c in reactor volume V^L^ are to be determined by FTIR/MCR-ALS. For MCR execution, $m_{i}^{C,L}$ must be split into one term containing the required concentrations from FTIR ex situ online measurement (ex) and into another term containing carbon compounds with concentrations accessible by in situ online measurement (on). For calculation of the online term, biomass concentration is observed by turbidity measurement and a calibrated exponential model. Dissolved carbon dioxide concentration is estimated by a soft-sensor based on Henry’s law and the CO_2_ mole fraction measured by a gas sensor in exhaust gas flow [35].

$$m_{i}^{C,L}=m_{i}^{C,L,\mathrm{on}}+m_{i}^{C,L,\mathrm{at}}$$

$$m_{i}^{C,L,\mathrm{on}}=\left(\alpha^{C,\mathrm{cell}}{\;c}_{i}^{\mathrm{cell},L}+\alpha^{C,{\mathrm{CO}}_{2}}{\;c}_{i}^{{\mathrm{CO}}_{2},L}\right)\;V_{i}^{L}$$

$$m_{i}^{C,L,\mathrm{ex}}=(\alpha^{C,\mathrm{glc}}{\;c}_{i}^{\mathrm{glc},L}+\alpha^{C,\mathrm{ace}}{\;c}_{i}^{\mathrm{ace},L}){\;V}_{i}^{L}$$

The ex situ online term ex, containing the concentrations to estimate by MCR, must be converted concerning the left side of inequality constraint $Ac_{i}^{T}\leq b_{i}$. Thus, the (2,Ω)-matrix **A** contains in the first row, on positions associated with concentrations of glucose and acetate, the fractions $\alpha^{C,\mathrm{glc}}$ and $\alpha^{C,\mathrm{ac}}$ multiplied with reactor volumes $V_{i}^{L}$. The first row is associated with the upper bounds $b^{u}$ of the constraint. The second row is the negative of the first row and is associated with the lower bounds $b^{l}$.

All other brought-in and recovery terms that are directly or indirectly accessible by online process sensors and soft-sensors, but not by FTIR/MCR-ALS, are used to form the **b**_i_ vector.

The carbon in the exhaust gas phase is calculated by the CO_2_ removal rate Q^CO2^, which in turn is calculated based on measurements of CO_2_ mass flow at gas phase entry and of inert gas balance to estimate exit mass flow.

$$m_{i}^{C,g}={\;\alpha }^{C,{\mathrm{CO}}_{2}}\overset{i}{\underset{1}{\int }}V_{i}^{L}\left(t\right)Q_{i}^{{\mathrm{CO}}_{2}}\left(t\right)\mathrm{dt}$$

The calculation of carbon in samples of fermentation media starts at the first FTIR observation i = 1 with known initial media concentrations $c_{0}$ and sample volumes $\Delta V_{0}^{\mathrm{scc}}$ and $\Delta V_{0}^{\mathrm{scf}}$ taken before the first FTIR measurement is observed. A certain error in sample carbon mass calculation must be accepted since the respective current values of $c_{i}^{\mathrm{glc},L}$ and $c_{i}^{\mathrm{ace},L}$ are unknown at the time of constraint calculation. Hence, at i > 1, the results of the last MCR step i-1 are utilised. Considering comparative slow bioprocess kinetics and a higher sampling frequency, this is a reasonable approximation.

$$m_{i}^{C,\mathrm{scc}}=\overset{m}{\underset{i=1}{\sum }}\left(\alpha^{C,\mathrm{glc}}c_{i-1}^{\mathrm{glc},L}+{\;\alpha }^{C,\mathrm{ace}}c_{i-1}^{\mathrm{ace},L}+{\;\alpha }^{C,\mathrm{cell}}c_{i-1}^{\mathrm{cell},L}+{\;\alpha }^{C,{\mathrm{CO}}_{2}}c_{i-1}^{{\mathrm{CO}}_{2},L}\;\right)\Delta V_{i-1}^{\mathrm{scc}}$$

$$m_{i}^{C,\mathrm{scf}}=\overset{m}{\underset{i=1}{\sum }}\left(\alpha^{C,\mathrm{glc}}c_{i-1}^{\mathrm{glc},L}+{\;\alpha }^{C,\mathrm{ace}}c_{i-1}^{\mathrm{ace},L}+{\;\alpha }^{C,{\mathrm{CO}}_{2}}c_{i-1}^{{\mathrm{CO}}_{2},L}\;\right)\Delta V_{i-1}^{\mathrm{scf}}$$

The brought-in carbon is the sum of the carbon fractions of glucose, acetate and cell mass in the initial medium as well as the supplied glucose from the feed reservoir.

$$m_{i}^{C,\mathrm{in}}=m_{i=1}^{C,L}+m_{i}^{C,r}=\left(\alpha^{C,\mathrm{glc}}{\;c}_{i=1}^{\mathrm{glc},L}+\alpha^{C,\mathrm{ace}}c_{i=1}^{\mathrm{ace},L}+\alpha^{C,\mathrm{cell}}c_{i=1}^{\mathrm{cell},L}\right)V_{i=1}^{L}+\alpha^{C,\mathrm{glc}}{\;c}_{i}^{\mathrm{glc},r}V_{i}^{r}$$

By that information, **b**_i_ can be calculated as

$$b_{i}=\left(\begin{array}{c} b^{u}\cdot m_{i}^{C,\mathrm{in}}-\left(m_{i}^{C,L,\mathrm{on}}+m_{i}^{C,g}+m_{i}^{C,\mathrm{scc}}+m_{i}^{C,\mathrm{scf}}\right)\\ -b^{l}\cdot m_{i}^{C,\mathrm{in}}+\left(m_{i}^{C,L,\mathrm{on}}+m_{i}^{C,g}+m_{i}^{C,\mathrm{scc}}+m_{i}^{C,\mathrm{scf}}\right) \end{array}\right)$$

The settings of the upper and lower tolerance bounds b^u^ and b^l^ of the carbon balance constraint are based on different considerations. Recovery rates higher than 1 are only caused by measurement errors while values below 1 are caused by both, measurement errors and not identified by-products. Therefore, the upper bound can be set tighter than the lower, with values in an interval of b^u^ = (0.9, 1.1), depending on the process phase. An upper bound lower than 1 may be suitable if it is evident that the carbon compounds which are considered in the constraint calculation but which lie outside the optimisation of **Ĉ** are underestimated (e.g., biomass). An upper bound higher than 1 is indicated if external carbon compounds seem to be overestimated. Thus, by tuning b^u^, it therefore is possible to compensate measurement errors in online sensor equipment. As mentioned above, the recovery rate can reach approximately 90% at the end of the *E. coli* process, although just based on glucose, acetate, CO_2_ and biomass. In order to take the formation of not considered carbon compounds into account, the lower bound is set in a defensive way to b^l^ = 0.8 to avoid over-restriction.

Furthermore, the non-negativity constraint is set for all concentration values, and at each new curve resolution step, the start value for lsqlin optimisation is set to the last estimation result. The initial concentration values for glucose, acetate, total phosphate and ammonia at i = 1 are set to the known batch medium concentration and may vary in a range of ±10%. 

Some online measurements such as fermenter weight, gas analysis and turbidity have a higher noise level and have to be filtered before further processing. Biomass estimated by turbidity is smoothed by application of an exponential smoothing filter with a smoothing factor alpha set to 0.05. The online signal of fermenter weight is prone to disturbances in form of high needle peaks, often caused by manual contact with the reactor e.g., while taking an offline sample. Those disturbances can easily be removed automatically by a threshold filter detecting differences between one measurement point to the next, higher than a threshold value e.g., >1.5 L, since offline samples usually have values below 1.5 and since the actual fermenter volume changing rate is much inferior. These few values in the sequel above the threshold are overwritten by the last value lower than the threshold. In this way, measurement errors can be significantly reduced since all concentration values are depending on the reactor volume. Outliers in the online gas analysis are treated correspondingly.

## 4. Results and Discussion

**Figure 3:**
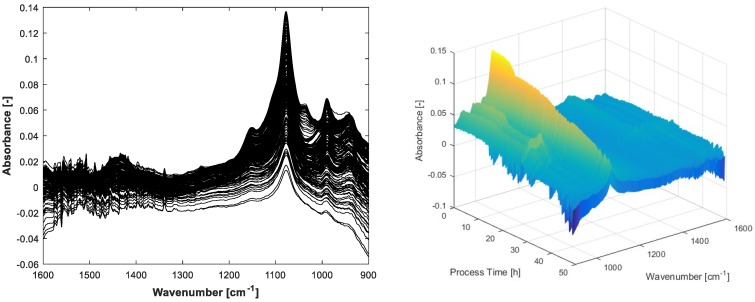
The 264 infrared spectra acquired from approximately 50 h fed-batch fermentation.

The carbon balance constraint algorithm with appropriate initial pure spectra estimations results in physically reasonable MCR solutions. By setting suitable start values and tolerance bounds, the rotatory and intensity ambiguities are reduced significantly. As a consequence, the concentration profiles of the substrates glucose, ammonia, total phosphate and the expected metabolic by-product acetate can be unfolded from the spectral mixture matrix **X** with minor manual measurement effort. An overview of the entire process spectra is displayed in Figure 3. 

The FTIR spectra show negative values because of the water background subtraction. The inflexions downwards on the left and right borders of the display are caused by air bubbles in the flow cell. These artefacts can be handled by MCR. Before integrating the spectral air bubble model in the MCR-ALS algorithm, the assumption for the spectral air bubble model was ascertained by concentration prediction of a simple aqueous solution containing glucose, acetate and phosphate. The solution was measured by FTIR with and without air on the ATR crystal surface. The prediction was executed by multiplying the measured spectra **X** with the pseudoinverse of **S**_0_, whereby **S**_0_ is a composition of pure spectra of known mixture components. In one experiment, **S**_0_ involves an estimated spectral model for air bubbles, in the other just the pure spectra of the solved components are compounded. As evident from Figure 2 and Table 2, the integration of the estimated air bubble signature results in a significant prediction improvement.

**Figure 4:**
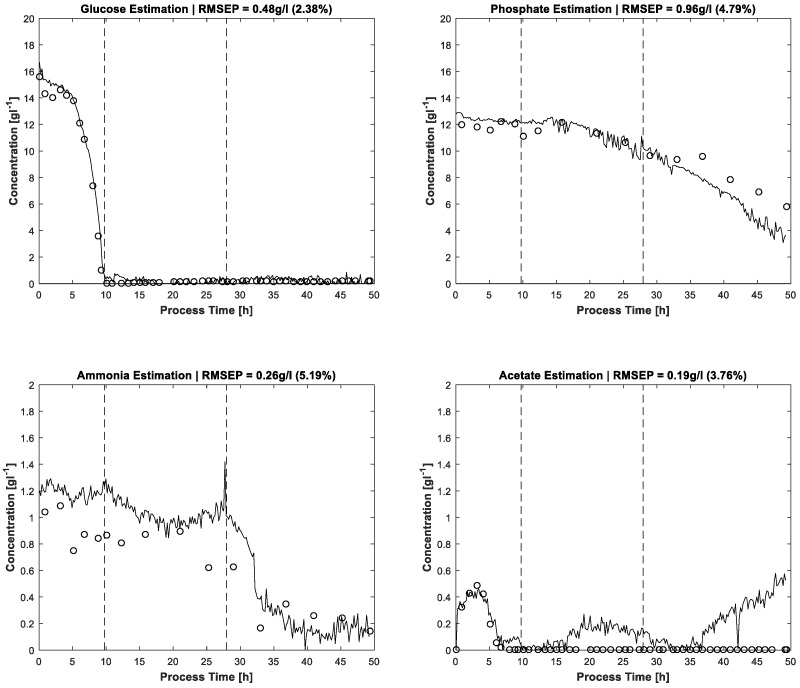
Concentration profiles estimated by FTIR/MCR-ALS (solid lines) and reference measurements (dots). The two dashed vertical lines in each plot distinguish the three process phases: batch phase (left), feeding phase/biomass production (middle), induction phase (right).

The results of MCR-ALS concentration prediction based on the 264 measured process spectra are shown in Figure 4. Elapsed calculation time for 300 ALS iterations was about 10 min on an Intel Core i7-4790 @3.6 GHz (4 Cores). It should be noted that, besides the constraints described above, just single manual measured spectra for each estimated pure component are utilised to achieve the resolution. Likewise, some of the pure component start values are just vectors of uniformly distributed random numbers. After 300 alternating least squares iterations, satisfactory approximations of the process dynamics are obtained. Glucose and phosphate are present in higher concentrations, so the resolution succeeds nearly without artefacts. At concentrations close to zero, a higher presence of artefacts and noise is expectedly obtained. Accordingly, the lower concentrated ammonium and acetate show a higher ratio of disturbances. 

The error evaluation takes place by comparing the FTIR/MCR-ALS concentration measurements with reference measurements. Concerning the residuals, the root mean squared errors (RMSE) are calculated and shown in Table 3.

The prediction results of the proposed MCR-ALS algorithm can be compared with prediction performances of PLS models. Acetate, ammonium and phosphate concentrations of a *Gluconacetobacter xylinus* fed-batch culture were predicted from spectra of in situ ATR-FTIR measurements by a PLS model with accuracies of 0.2, 0.17 and 0.24 gL^−1^, respectively [24]. The validation errors for offline samples of the same process were 0.22 gL^−1^ (acetate), 0.24 gL^−1^ (ammonium) and 0.18 gL^−1^ (phosphate). The applied PLS regression model is based on 56 mixture solutions, used as calibration standards. The accuracies of MCR-ALS estimation for ammonium and acetate are similar to the PLS errors of the referenced paper. The absolute error of phosphate prediction is higher for the MCR-ALS approach than for the described PLS method, the measurement range being about two times higher, too. In consideration of the minor calibration effort of the proposed MCR-ALS approach, the results are impressive. Furthermore, the PLS glucose prediction accuracy by at-line ATR-FTIR monitoring of an antibiotic fermentation process is with 0.56 gL^−1^ similar to the present prediction by MCR-ALS [23]. The PLS calibration model for glucose is based on 70 filtrated fermentation samples. Here, too, the reduction of calibration effort by effective online sensor data usage is evident when compared to PLS.

**Figure 5:**
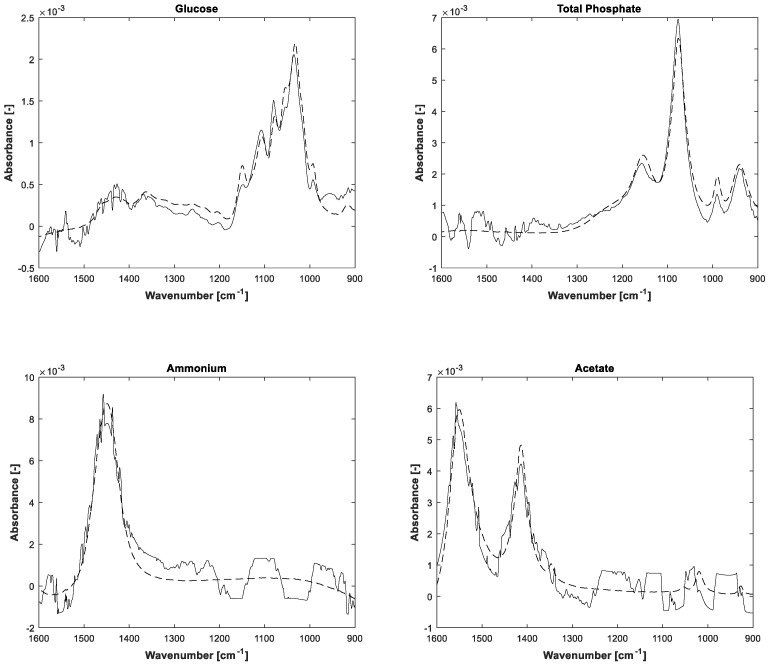
Initial measurements of pure substances (dashed lines) and identified pure spectral components after 300 ALS iterations (solid lines).

The estimations of pure component spectra are displayed in Figure 5. In addition to the notice concerning the associated concentrations, the higher noise level of the lower concentrated ammonium and acetate is also apparent in the pure spectral components.

**Figure 6:**
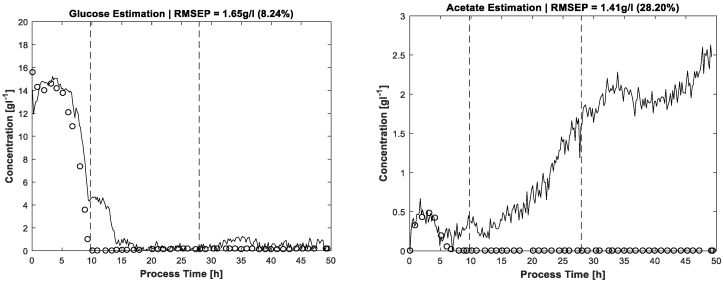
Concentration profiles for glucose (left) and acetate (right), estimated without application of the carbon balance constraint.

By way of comparison, Figure 6 shows the results for glucose and acetate concentrations without application of the carbon balance constraint but including the same constraints as used for pure spectra estimation, see above. Between hour 10 and 15 there is a significant artefact observable in the glucose concentration profile. The concentration estimation is too high, also discernible by carbon recovery rates approaching almost 1.2 in this process phase. A second drift in glucose concentration is located around t = 35 h without obvious reflection in carbon balance because of the lower deviation. In any case, without the carbon balance constraint, the solution space of MCR is enlarged, thereby also increasing the risk of ambiguities which can cause physically nonsensical solutions. For the same reason, the acetate profile in Figure 6 gives the impression of increasing concentrations which actually are not present. Nevertheless, the shapes of the actual existing concentration profiles in the batch phase are more or less recognised, the artefacts increasing mostly in the respective zero-concentration phases.

**Figure 7:**
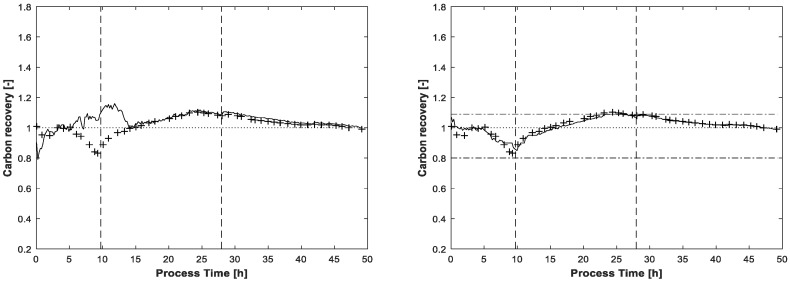
Carbon recovery based on the final glucose and acetate estimations after 300 ALS iterations, without (left) and with (right) application of the carbon balance constraint (solid line). Carbon recovery calculated by offline determined glucose and acetate concentrations (+). The dot-dashed horizontal lines indicate the highest upper and lowest lower bounds, only enabled in the run displayed on the right. The dotted horizontal line is the theoretical recovery rate of 1. The vertical lines indicate the three process phases: batch, feeding and induction.

The carbon recovery at the end of the MCR-ALS procedure is shown in Figure 7. Without application of the carbon balance constraint, the recovery rate exceeds two times the value of 1.09, once in the beginning and once again at the end of fed-batch phase. Even the lower value of 0.8 is slightly undershot at the beginning of the process. Around t = 25h, near the end of the feeding phase, the recovery rate with the enabled carbon balance constraint touches the highest upper bound of 1.09. As for the final estimated concentrations, the lower bound of 0.8 is not reached.

## 5. Conclusions

This study has shown that MCR-ALS with tailored constraints is capable of analysing simultaneously the concentrations of glucose, acetate, ammonium and total phosphate from ex situ online recorded FTIR spectra of an *E. coli* HCDC fermentation process. The required concentration information, extracted from 264 FTIR spectra and recorded over 50 h process time, has been estimated in accuracies between 0.19 and 0.96 gL^−1^. These results are comparable to established concentration estimations by PLS models, but are achieved with less calibration effort. It became apparent that the application of appropriate constraints, in particular the carbon balance constraint, improves the accuracy of concentration estimation in the ALS solution process by avoiding artefacts caused by rotatory ambiguities. In MCR-ALS concentration estimation, the carbon mass balance constraint, calculated by online sensor data, reduced ambiguities in glucose and acetate concentrations significantly. In pure spectra estimation, initial FTIR measurements of the required analytes as well as a spectral air bubble model led to appropriate MCR solutions. Besides the automatically sampled online FTIR spectra, all applied constraints are calculated broadly based on automated measurements and analytical process knowledge. It is shown that by introducing prior knowledge and processed non-spectroscopic online sensor data into the ALS procedure, better spectra resolution performances as well as efficient fermentation process analysis can be achieved.

## Figures and Tables

**Table 1:** Columns in ${\hat{S}}_{0}$ with initial spectra and constraint settings.

	(ŝ_0_)_1_	(ŝ_0_)_2_	(ŝ_0_)_3_	(ŝ_0_)_4_	(ŝ_0_)_5_	(ŝ_0_)_6_	(ŝ_0_)_7_	(ŝ_0_)_8_	(ŝ_0_)_9_	(ŝ_0_)_10_	(ŝ_0_)_11_	(ŝ_0_)_12_
**pure**	glc	ace	NH_4_^+^	H_2_PO_4_^−^ HPO_4_^2−^	IPTG	MgSO_4_	air	H_2_O	citric acid	unifrnd	unifrnd	unifrnd
**u**	0.1	0.1	0.1	0.1	0.1	0.1	inf	inf	0.1	inf	inf	inf
**l**	−0.1	−0.1	−0.1	−0.1	−0.1	−0.1	−inf	−inf	−0.1	−inf	−inf	−inf

Initial pure components: glucose (glc); acetate (ace); isopropyl β-d-1-thiogalactopyranoside (IPTG); air bubbles estimation (air); uniformly distributed random values (unifrnd); infinity (inf).

**Table 2:** Concentration estimation by multiplying known mixture spectra with pseudo inverses of pure spectral component matrices with and without integration of an air bubble model.

	Air Bubble Model in S_0_	Glucose [gL^−1^]	Ammonium [gL^−1^]	Phosphate [gL^−1^]
**Medium with air bubbles**	No	9.65	0	6.17
	Yes	15.63	0.52	7.70
**Medium without air bubbles**	No	16.56	0.57	8.16
	Yes	16.32	0.55	8.10
**Known concentration**	-	15	0.7	8

**Table 3:** Prediction performance: error estimation quantified by root mean squared distances between FTIR-MCR-ALS predictions and reference measurements.

Substance	RMSE [gL^−1^]	rel. RMSE [%]	Expected Range [gL^−1^]
Glucose	0.48	2.38	(0, 20)
Phosphate	0.96	4.79	(0, 20)
Ammonium	0.26	5.19	(0, 5)
Acetate	0.19	3.76	(0, 5)

Root mean squared error (RMSE); relative RMSE related to expected range (rel. RMSE).
